# Effectiveness of a nurse-delivered (FOCUS+) and a web-based (iFOCUS) psychoeducational intervention for people with advanced cancer and their family caregivers (DIAdIC): study protocol for an international randomized controlled trial

**DOI:** 10.1186/s12904-021-00895-z

**Published:** 2021-12-28

**Authors:** Orphé Matthys, Aline De Vleminck, Sigrid Dierickx, Luc Deliens, Vincent Van Goethem, Lore Lapeire, Mogens Groenvold, Line Lund, Caroline Moeller Arnfeldt, Lisa Sengeloev, Helle Pappot, Anna Thit Johnsen, Suzanne Guerin, Philip J. Larkin, Catherine Jordan, Michael Connolly, Paul D’Alton, Massimo Costantini, Silvia Di Leo, Monica Guberti, Elena Turola, Agnes van der Heide, Erika Witkamp, Judith Rietjens, Maaike van der Wel, Kevin Brazil, Gillian Prue, Joanne Reid, David Scott, Katherine Bristowe, Richard Harding, Charles Normand, Peter May, Catherine Cronin, Laurel Northouse, Peter Hudson, Joachim Cohen

**Affiliations:** 1 End-of-life Care Research Group, Vrije Universiteit Brussel (VUB) & Ghent University, Brussels, Belgium; 2 Department of Medical Oncology, Ghent University Hospital, Ghent, Belgium; 3 Department of Public Health, University of Copenhagen and Palliative Care Research Unit, Bispebjerg and Frederiksberg Hospital, Copenhagen, Denmark; 4 Department of Oncology, Herlev Gentofte University Hospital, Herlev, Denmark; 5 Department of Oncology, Rigshospitalet, University of Copenhagen, Copenhagen, Denmark; 6 University of Southern Denmark, Odense, Denmark; 7 UCD School of Psychology, University College Dublin, Belfield, Ireland; 8 Palliative and Supportive Care Service, Chair of Palliative Care Nursing, Lausanne University Hospital and University of Lausanne, Lausanne, Switzerland; 9 School of Nursing, Midwifery and Health Systems, University College Dublin, Dublin, Ireland; 10 Scientific Directorate, Azienda USL-IRCCS di Reggio Emilia, Reggio Emilia, Italy; 11 Psycho-oncology Unit, Azienda USL - IRCCS di Reggio Emilia, Reggio Emilia, Italy; 12 Nursing & Health Care Professions Directorate, Azienda USL-IRCCS, Reggio Emilia, Italy; 13 Deparmtent of Public Health, Erasmus MC, University Medical Center Rotterdam, Rotterdam, the Netherlands; 14 School of Nursing and Midwifery, Queen’s University of Belfast, 97 Lisburn Road, Belfast, Northern Ireland BT9 7BL UK; 15 King’s College London, Florence Nightingale Faculty of Nursing Midwifery and Palliative Care, Cicely Saunders Institute, London, UK; 16 Centre for Health Policy and Management, Trinity College Dublin, Dublin, Ireland; 17 Cicely Saunders Institute of Palliative Care, Policy and Rehabilitation, London, UK; 18 The Irish Longitudinal Study on Ageing, Dublin, Ireland; 19 School of Nursing, University of Michigan, Ann Arbor, MI USA; 20 Centre for Palliative Care, St Vincent’s Hospital, Melbourne, Australia; 21 Vrije University Brussels (VUB), Brussels, Belgium

**Keywords:** Psychoeducational intervention, Dyadic, cancer, Family caregiver, Randomized clinical trial

## Abstract

**Background:**

Worldwide, millions of people with advanced cancer and their family caregivers are experiencing physical and psychological distress. Psychosocial support and education can reduce distress and prevent avoidable healthcare resource use. To date, we lack knowledge from large-scale studies on which interventions generate positive outcomes for people with cancer and their informal caregivers’ quality of life. This protocol describes the DIAdIC study that will evaluate the effectiveness of two psychosocial and educational interventions aimed at improving patient-family caregiver dyads’ emotional functioning and self-efficacy.

**Methods:**

We will conduct an international multicenter three-arm randomized controlled trial in Belgium, Denmark, Ireland, Italy, The Netherlands, and the United Kingdom. In each country, 156 dyads (936 in total) of people with advanced cancer and their family caregiver will be randomized to one of the study arms: 1) a nurse-led face-to-face intervention (FOCUS+), 2) a web-based intervention (iFOCUS) or 3) a control group (care as usual). The two interventions offer tailored psychoeducational support for patient-family caregiver dyads. The nurse-led face-to-face intervention consists of two home visits and one online video session and the web-based intervention is completed independently by the patient-family caregiver dyad in four online sessions. The interventions are based on the FOCUS intervention, developed in the USA, that addresses five core components: family involvement, optimistic outlook, coping effectiveness, uncertainty reduction, and symptom management. The FOCUS intervention will be adapted to the European context. The primary outcomes are emotional functioning and self-efficacy of the patient and the family caregiver, respectively. The secondary outcomes are quality of life, benefits of illness, coping, dyadic communication, and ways of giving support of the patient and family caregiver.

**Discussion:**

DIAdIC aims to develop cost-effective interventions that integrate principles of early palliative care into standard care. The cross-country setup in six European countries allows for comparison of effectiveness of the interventions in different healthcare systems across Europe. By focusing on empowerment of the person with cancer and their family caregiver, the results of this RCT can contribute to the search for cost-effective novel interventions that can relieve constraints on professional healthcare.

**Trial registration:**

Registration on ClinicalTrials.gov on 12/11/2020, identifier NCT04626349.

**Date and version identifier:**

20211209_DIAdIC_Protocol_Article.

**Supplementary Information:**

The online version contains supplementary material available at 10.1186/s12904-021-00895-z.

## Background

### Background and rationale

Each year, 3.7 million people in Europe are diagnosed with cancer, accounting for 1.9 million deaths [1]. The symptoms and burden of cancer patients range from anxiety and depression to pain and fatigue [2]. Something that is often overlooked is that the effects of cancer extend from patients to their family caregivers. A family caregiver is an unpaid, informal provider of care who has a personal connection to the patient (a friend, partner, ex-partner, sibling, parent, child, or other blood or non-blood relative) and provides one or more physical, social, practical, and emotional tasks [3]. The caregiving role they take on can result in severe physical, psychological, and emotional health problems (co-suffering) and an overall decline in quality of life (QoL), which has major public healthcare and health resource implications [4]. Taking into account an expected rise in the number of people with cancer, a significant increase in the ageing population, governments’ efforts to limit the healthcare spending, and shortages of professional health caregivers, it is likely that the future need for adequate care in the population will increase dramatically and cannot be borne entirely by formal healthcare. At the same time, the population of unpaid caregivers is also becoming older and frailer, increasing the need to support their efforts.

Despite the serious problems that cancer creates for patients and their family caregivers, there are only a few interventions that target both patient and family caregiver to self-manage the effects of the advanced phase of the illness [5–7]. Literature shows that dyadic interventions, i.e. targeting the patient and family caregiver together, are more likely to result in better outcomes for both parties and are more cost-effective than single target interventions [8]. Interventions focusing on the QOL of both the patient and the family caregiver (i.e. the dyad) may promote their well-being, lessen their burden, and reduce the economic toll of advanced cancer care. By focusing on the empowerment of the dyad, pressure on professional care providers may also be reduced.

Currently, we lack the evidence to recommend which psychosocial and educational interventions, provided to both patients and their family caregivers will generate the most favourable outcomes for both. To select the most promising dyadic interventions to examine, the DIAdIC research consortium has assessed existing interventions that support patients with advanced cancer and their family caregivers, with a potential fit for Europe and that are relevant to the health objectives of the European Commission as outlined in the white paper “Together for Health: A Strategic Approach for the EU 2008-2013”. The FOCUS intervention developed in the USA by Northouse et al. [5] was identified as among the most effective and potentially relevant. The face-to-face FOCUS program was developed as a nurse-delivered face-to-face intervention offering information and support to both patients and family caregivers. Three randomized controlled trials (RCTs) in the USA demonstrated improved QoL and well-being of both patients and caregivers [5, 7, 9]. The dyads also reported significantly less negative appraisal of illness and caregiving, less uncertainty and hopelessness, improved communication within dyads, and improved caregiver self-efficacy compared to control dyads. To make the FOCUS program available to more people, the core component (increasing family involvement) of the face-to-face program was later translated into a tailored, web-based format [6]. For the web-based FOCUS intervention, significant effects were found in a pre-post study on dyads’ QoL, emotional distress, perceived benefits of illness/caregiving, and caregivers’ self-efficacy. The option of delivering a psychoeducational intervention via a computer program fits well in the era of telemedicine. The importance of providing support remotely is currently highlighted during the COVID-19 pandemic.

The existing FOCUS interventions from the USA have been substantially tailored, updated, and adapted by the DIAdIC research consortium to meet the needs of patients with advanced cancer and their family caregivers in six European countries. The core components of the face-to-face program have been translated into a web-based format. These adaptations and updates have led to the development of two new interventions: 1) the face-to-face FOCUS+ intervention and 2) the iFOCUS web intervention. The adaptation process will be reported elsewhere.

The effectiveness, cost-effectiveness, and mechanisms of action of both interventions will be investigated in a large-scale international randomized controlled trial (RCT). The current manuscript aims to present the research protocol of this RCT study. The Standard Protocol Items: Recommendations for Interventional Trials (SPIRIT) statement was applied to describe all relevant aspects of the trial.

#### Objectives

The overall aim of this project is to evaluate the effectiveness, cost-effectiveness, and mechanisms of action of two psychoeducational interventions (a face-to-face nurse-led intervention called FOCUS+ and a web-based intervention called iFOCUS) aimed at improving the emotional functioning and self-efficacy of patients with advanced cancer and their family caregiver. Both interventions are compared to care as usual.

##### Project objectives

1. To compare 1) the face-to-face FOCUS+ intervention and 2) the iFOCUS web-based intervention to 3) care as usual in terms of their:
  Effect on the emotional functioning and self-efficacy (primary outcomes), appraisal of illness, uncertainty, hopelessness, coping, dyad communication, QoL, and healthcare resource use of patients with advanced cancer and their family caregivers
  Cost-effectiveness, taking into account the use of the formal health system, unpaid care burden, and QoL
  Effects on vulnerable subgroups (particularly women and those of lower socioeconomic status)
  Effectiveness in different healthcare systems
2. To evaluate the implementation process of the interventions in terms of the acceptability, feasibility, usefulness as perceived by patients, family caregivers, and healthcare staff in each country, and their mechanisms of action.

#### Trial design

This study is an international multicenter parallel-group three-arm superiority trial comparing 1) the FOCUS+ face-to-face intervention (intervention 1) and 2) the iFOCUS web-based intervention (intervention 2) to 3) standard care (control group).

We use a parallel-group design meaning that each group receives either intervention 1, intervention 2, or standard care as usual. As the risk of contamination is limited, an individual RCT rather than a cluster RCT is feasible and more appropriate.

Randomization will be performed per a computer randomization schedule with a 1:1:1 allocation ratio to one of the two intervention arms or the standard care arm (control group).

## Methods: participants, interventions, and outcomes

### Study setting

Both interventions (FOCUS+ and iFOCUS) will be administered in the homes of the patient-caregiver dyads (or in the location of the dyad’s preference).

The interventions will be conducted in six countries (Belgium, Denmark, Ireland, Italy, the Netherlands, and the United Kingdom). The selection of countries is based on a number of considerations: (1) feasibility and capacity to conduct a large-scale complex trial in the targeted population, (2) a variation in healthcare and welfare system typologies (Bismarck, Beveridge, Social-Democrat systems [10]), (3) regional variation across Europe (Northern, Western, Southern) and (4) a relatively advanced level of palliative care development and integration within oncology care.

### Eligibility criteria

#### Participants

The study population will consist of patients with advanced solid organ cancer (except brain cancer) and their primary family caregiver (as determined by the patient). The inclusion and exclusion criteria for both the patient and the family caregiver are described in Table 1. Patients with brain cancer are excluded as they may experience difficulty completing the intervention and questionnaires due to cognitive issues. Patients with a prognosis of fewer than 3 months are also excluded as they may be too vulnerable, and the interventions are tested over 3 months.

**Table 1:** Inclusion and exclusion criteria for patients and family caregivers

Inclusion criteria	*Screened by*	Exclusion criteria	*Screened by*
Patient			
Diagnosis of cancer: solid organ (lung, colorectal, breast, prostate, and other)	Treating clinician or RA*	Brain cancer, non-solid cancers	Treating clinician or RA
No longer receives curative treatment (only life-prolonging or palliative treatments)	Treating clinician or RA	Prognosis of fewer than 3 months	Treating clinician or RA
Treating clinician would not be surprised if the patient died within 2 years [11]	Treating clinician or RA	Has no family caregivers	RA
Written informed consent	RA	< 18 years old	RA
Lives within feasible distance for intervention nurses to travel	RA	Unable to participate in available languages	RA
Family caregiver			
Written informed consent	RA	Unable to physically or mentally participate	RA
Primary family caregiver as determined by the patient	RA	Cancer diagnosis in the last 12 months	RA
Lives within feasible distance for intervention nurses to travel	RA	< 18 years old	RA
		Unable to participate in available languages	RA
Dyad			
Patient and/or family caregivers have access to and are familiar with the use of the internet	RA		

RA* = research assistant, either from the study team or in situ

Previous studies of FOCUS interventions in the USA have found the interventions to be feasible and effective in a variety of solid cancer types as the interventions are tailored to the specific cancer type and symptoms patients experience [4, 8].

#### Study centers

In each country, patients with advanced cancer and their primary family caregiver will be recruited and enrolled via participating hospitals. Inclusion criteria for hospitals participating in this study are that they 1) treat patients with advanced cancer and 2) deliver oncology care. The first selection of hospitals and departments within these hospitals (e.g. oncology, pneumology) is informed by the number of individual patients seen per year meeting the eligibility criteria, to ensure that we will be able to include sufficient patients to meet the requirements for statistical power.

#### Nurses performing the FOCUS+ face-to-face intervention

Two to four nurses per country will be hired on the project to conduct the FOCUS+ face-to-face intervention. They will be able to communicate in the language appropriate for their country and must be sufficiently proficient in English to be able to participate in the study training which will be delivered in English. They will have a professional nursing qualification as recognized in each participating country. The nurses will have significant clinical experience with people with advanced cancer and/or palliative care. An overview of the qualification criteria for intervention nurses is provided in Table 2.

**Table 2:** Nurse entry-level skills

- Communicate in a language appropriate to their country - Sufficient proficiency in English to be able to participate in the training - A professional nursing qualification recognized in each participating country - Experience in advanced cancer care or palliative care - Excellent communication skills - Perceptive listening and questioning skills - Ability to cope with emotionally demanding situations - Willingness to work flexibly - Desirable criteria: a post-graduate qualification in nursing	

### Interventions and control

This study comprises two interventions: the FOCUS+ face-to-face intervention and the iFOCUS web-based intervention. Both interventions are delivered in addition to usual care. Both interventions are psychoeducational interventions focusing on teaching dyads optimal ways to jointly manage the implications of advanced cancer and responding to their priority concerns.

The theoretical framework behind the interventions is the transactional model of stress and coping of Lazarus and Folkman [12]. In this model, stress is produced by an individual’s response to stressors in their environment and the response of an individual to these stressors. In the context of advanced cancer, a series of personal, social, and illness-related factors (antecedents) influence how patients and caregivers appraise the illness and cope with the demands associated with it. Previous studies have shown that these antecedents are significant predictors of psychosocial outcomes in cancer patients and their family caregivers [13].

The interventions are designed to be tailored to the specific needs and wishes of the patient-caregiver dyads. The tailoring is based on the information from the baseline measures and the responses in the intervention sessions. Both interventions aim to enhance dyads’ emotional functioning and their self-efficacy.

The FOCUS+ and iFOCUS interventions have different modes of administration (face-to-face vs. web-based) but have the same core content by addressing five core components: (1) supporting family involvement, communication, and mutual support, (2) supporting outlook and meaning, (3) increasing coping effectiveness, (4) reducing uncertainty and (5) teaching symptom management and giving them the confidence to handle specific tasks and problems. These five core components stem from the original FOCUS intervention [5] developed in the USA and have been translated and adapted to the current European context (Table 3).

**Table 3:** Five conceptual core components of the FOCUS+ and iFOCUS interventions

Core concept	Goals
Supporting family involvement, communication, and mutual communication (F)	- Discuss and support communication - Encourage mutual support and teamwork in a planned program of care - Identify family strengths - Help children in the family as needed
Supporting outlook and meaning (O)	- Help dyads share fears and concerns - Discuss positive and negative feelings of dyads - Educate dyads about different kind of feelings and attitudes - Encourage dyads to set realistic short-term goals
Increasing coping effectiveness (C)	- Help dyads deal with overwhelming stress - Discuss and support active coping strategies by dyads - Assist caregivers to manage the demands of illness
Reducing uncertainty (U)	- Educate dyads about disease and treatments as needed - Teach dyads how they can obtain additional information - Help dyads learn ways to live with uncertainty
Teaching symptom management and giving the confidence to handle specific tasks and problems (S)	- Assess symptoms in patients and family caregiver - Teach self-care strategies to manage symptoms (e.g. ways to manage reactions and side effects associated with the illness, treatments, and adjustment) - Help dyads identify relevant resources in the community (community services and support)

#### Face-to-face intervention (FOCUS+)

**Fig. 1:**
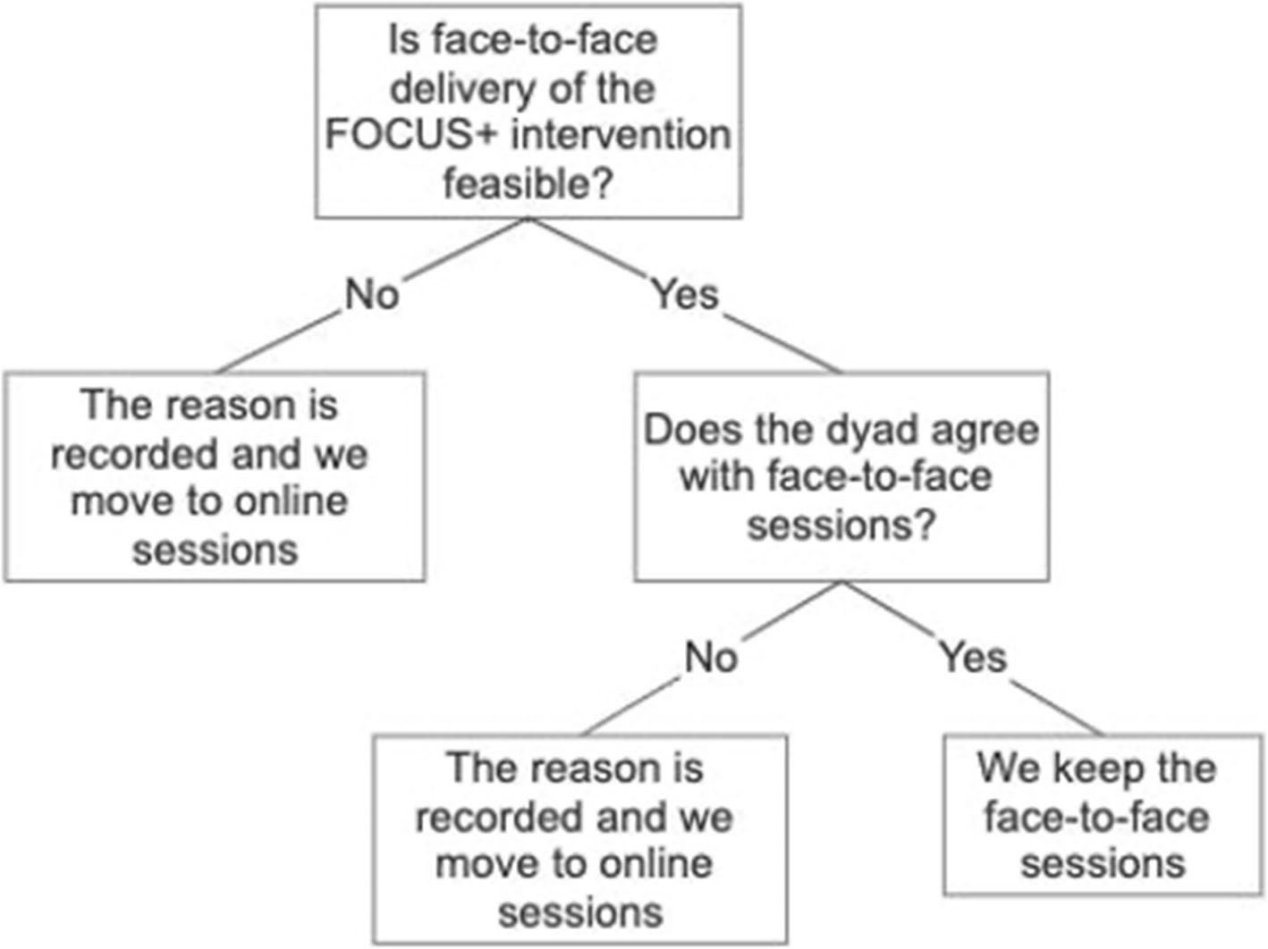
Decision-making process in method of delivery of FOCUS+ intervention in light of possible COVID-19 developments

**Fig. 2:**
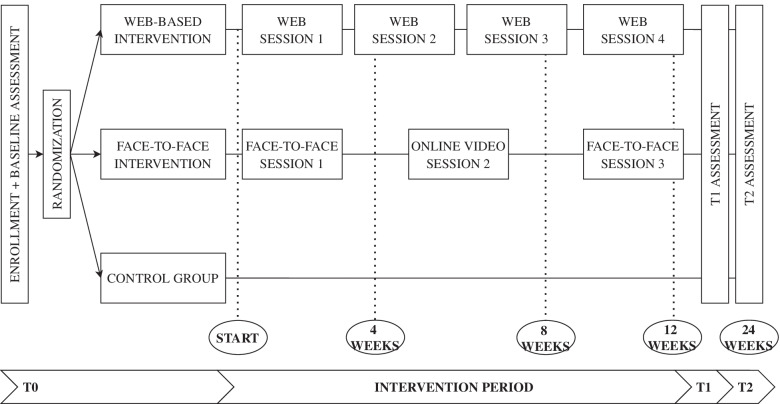
Participant timeline

The face-to-face FOCUS+ intervention is a home-based intervention consisting of two 90-min home visits and one 30-min online video session, conducted by a trained intervention nurse over 12 weeks, focusing on the five core components. An overview of the participant timeline can be found in Fig. 2. In case of tightened measures to contain the COVID-19 pandemic, the delivery of the FOCUS+ sessions via a GDPR-approved online platform (e.g. MS Teams, Zoom,..) will be allowed, if necessary. The decision-making process is visualized in Fig. 1 and will be recorded for each dyad in each country.

The nurses will receive extensive online training (including both synchronous and asynchronous training modules) and additional continuous follow-up training to provide them with the knowledge and skills required to successfully implement the intervention. The specific learning outcomes of the training are: (1) Understand the overall aims and objectives of the intervention, (2) Demonstrate understanding of the principles of FOCUS+ and the need to support both the patient and carer together, (3) Prepare for, conduct and complete the intervention, including the delivery of the intervention through online sessions with the dyads if needed and (4) Demonstrate how to use the intervention manual and other identified materials specific to the nurses’ country. The training will be interactive. This means that the participating nurses will have the opportunity to share their experience and insights and considerable time will be spent reviewing the developed nurse intervention manual and discussing the intervention fidelity. Nurses will also be asked to review and analyze case studies, engage in reflection and discussion regarding training content, and will be invited to discuss potential barriers and facilitators to implementing the intervention in their home countries. The synchronic online training sessions will be video recorded to give participating nurses the opportunity to review the material as needed and to facilitate the training of new nurses who may join the study over time.

The intervention is comprehensively manualized with a checklist-format and there is a protocol to guide the delivery of the intervention for each home visit and online video session, including strategies to respond to relevant issues with the patient and their family caregiver.

Adherence to, and fidelity of, the intervention is monitored by the use of the fidelity checklist. As part of the face-to-face intervention, it is proposed that all intervention sessions are audio-recorded to allow assessment of fidelity for a stratified random selection (20%) of dyads. A stratified random sample will be used to ensure that objective fidelity checks are performed across the timeline of the study, allowing for the maintenance of fidelity to be assessed. As such, it is proposed that the 15-month timeframe for intervention delivery be divided into five three-month blocks, with 20% of completed sessions with dyads assessed in each block. For reflexive fidelity, intervention nurses will be asked to self-assess fidelity based on the recordings of the first of each of the three FOCUS+ sessions they complete. These fidelity checks will be conducted in the early phases of delivery (block 1), to allow for intervention nurses to adapt their practice if needed to ensure that appropriate levels of fidelity are met. These reflexive checks will also be conducted by a member of the research team, allowing for a discussion between the intervention nurses and the researchers on the implementation of the intervention. Following these early reflexive fidelity checks, nurses will receive reports of fidelity findings every 3 months to support ongoing reflection on the consistency of intervention delivery.

A printed FOCUS+ guide will be handed out to the dyads providing practical information, concrete tips, and advice for persons with advanced cancer and their family caregiver. In addition, more detailed national cancer-related information via brochures, leaflets, and websites will be made available to the dyad, based on their needs. Both the FOCUS+ guide and the additional brochures and information leaflets serve as additional supporting resources for reference between or after the sessions.

By taking into account the dyad’s comments and questions during the sessions, the content of the sessions is always tailored to the specific needs of the dyads. The nurse can spend more time on components that can be of particular use to the dyad, or some content areas can be discussed minimally if the dyad does not require assistance in a particular area.

#### Web-based intervention (iFOCUS)

The web-based iFOCUS intervention is a self-managed intervention that is completed autonomously by the patient-caregiver dyads together in their home (or a location of their preference). It encompasses four sessions spread over 12 weeks, focusing on the same five core components as the face-to-face intervention. The sessions are completed simultaneously by the patient and the family caregiver, sitting side by side at a computer or tablet. Access to the sessions will be provided via a link sent by email. All sessions of the iFOCUS intervention are available in the official language of the participating countries.

Similar to FOCUS+, except fully automated by a web-program, dyads are asked to assess their strengths, problems, and needs, and based on these assessments tailored educational content is presented to the dyad. The FOCUS+ guide from the face-to-face intervention will be replaced by an online personal workbook. It contains the results of the interactive exercises that are provided to the dyads during the web-sessions, supplemented with related information from the FOCUS+ guide and links to other cancer information brochures, leaflets, and websites. A helpdesk is available (via email or telephone) for dyads doing the web-sessions to resolve any technical difficulties in each country.

Routine data from the web-platform will be accessed by a member of the research team to allow for fidelity assessment. Mirroring the FOCUS+ fidelity checklist, this will assess both completion of key iFOCUS elements and the time spent by dyads on each of the five (F O C U S) components.

The content of the iFOCUS intervention will also be tailored to their demographics (age, sex, and the relationship of the dyad) and information provided during the web-based sessions. The tailoring is done to enhance the relevance of the intervention content.

#### Control group (standard care as usual)

Patients in the control group will receive standard care as usual, as determined by the healthcare system in the participating countries. The dose and frequency of usual care will be deemed appropriate by the medical practitioner in charge of their treatment.

#### Criteria for discontinuing or modifying allocated interventions

When a patient or family caregiver dies during the study period, a bereavement protocol will be in place. The national trial manager will be notified about the death of a participant by either the intervention nurse (for the FOCUS+ intervention) or the data collector (for the FOCUS+ and iFOCUS intervention) who should be informed about this when trying to make an appointment with the dyad. The bereaved participant from the iFOCUS intervention will be able to inform the national trial manager about the passing of their relative via e-mail. For the FOCUS+ intervention, the bereaved participant will receive a phone call from the intervention nurse outlining condolences and the options for where they can access support (relevant to each country) if required and what the implications are for the study. For the iFOCUS intervention, the bereaved participant will receive a similar phone call from a research assistant appointed within each research team, that has relevant qualifications and/or significant clinical experience (e.g. a psychologist). If the bereaved person seems very distressed, the nurse or research assistant can offer to call the person a second time (e.g., 1 week later) to determine whether the person has made contact with a source of support (e.g., family, clergy, physician, bereavement group). Bereaved persons will not be asked or expected to complete follow-up study questionnaires but will be offered the option to continue to fill out an abbreviated package of follow-up questionnaires that are relevant to the remaining partner (e.g. emotional functioning and QoL scale). Any other reason for discontinuing the interventions is described in the informed consent (dyads can withdraw from the study at any moment) or under the section of adverse events (such as psychological distress).

#### Strategies to improve adherence to the intervention protocols

Strategies include:

- Nurses delivering the intervention will receive extensive training and additional continuous follow-up training to provide them with the knowledge and skills required to successfully implement the intervention.
- The FOCUS+ intervention is comprehensively manualized. Nurses will be trained in the use of an intervention manual to deliver the FOCUS+ intervention.
- Adherence and fidelity monitoring: nurses delivering the FOCUS+ intervention will be required to self-assess fidelity based on the recordings of the initial delivery of the three FOCUS+ sessions with dyads with whom they have carried out the intervention. These fidelity checks will be conducted in the early phases of delivery, to allow for intervention nurses to reflect on the initial experience of delivery. Following this early phase, intervention nurses will be provided with routine reports on overall fidelity checks to allow them to adapt their practice if needed to ensure appropriate levels of fidelity are met throughout delivery.
- It is proposed that 20% of dyads are assessed for objective fidelity using the intervention fidelity checklist by a member of the research team, with the dyad’s full intervention participation (that is all sessions of the intervention) assessed as one check.
- Discussing fidelity of FOCUS+: planned and unplanned adaptations in the interventions during the training of the nurses, the regular community of practice sessions with trainers and nurses, and the monthly follow-up sessions between the trainers and nurses (virtual meetings during the study period).
- A number of aspects of adherence are built into the iFOCUS web-module; e.g. during web-sessions dyads are asked to confirm that both of them are present and indicate where they are sitting at the computer.
- If dyads do not fully complete an iFOCUS web-session and exit the session before finishing, a reminder will be sent via email.
- Dyads who experience problems with onboarding for the web-sessions will receive help from the national trial manager with onboarding via a phone call.
- Routine monitoring will also include surveys of satisfaction with the interventions.

#### Relevant concomitant care and interventions that are permitted or prohibited during the trial

There are no restrictions regarding concomitant care during the trial outside of the three trial arms.

### Outcomes

#### Study endpoints and assessments

This study uses both qualitative and quantitative data to measure the outcomes of the intervention. Data will be collected three times from patient-caregiver dyads; (1) baseline measure before randomization to the study arms (T_0_) (2) first follow-up at 12 weeks (T_1_) and (3) second follow-up at 24 weeks (T_2_).

The primary endpoints are emotional functioning and self-efficacy of both the person with advanced cancer and the family caregiver at T_1_. The secondary endpoints are QoL of the patient and caregiver, benefits of illness, coping, dyadic communication, ways of giving support at T_1_. All listed primary and secondary outcomes are measured at T_2_ and formal healthcare use and costs are measured at T_1_ and T_2_.

Validated questionnaires will be used to measure the primary and secondary endpoints. In addition, data on socio-demographic characteristics, care received and participant perspectives on the acceptability, feasibility, and usefulness of the intervention will be collected. An overview of the instruments, the underlying measured concepts, and their timing can be found in Table 4. The complete questionnaire is added as a supplementary file.

**Table 4:** Instruments, underlying concepts, and timing

Concept	Measured by^b^	Timing^**a**^		
		T_0_ (Before randomization)	T_1_ (T_0_ + 12 weeks)	T_2_ (T_0_ + 24 weeks)
***Outcome measures used for the primary endpoints***				
**Emotional Functioning**	EORTC [14–16] 10 emotional functioning items described by Jabbarian et al. [15]. For **patients** (10 items): For **caregivers** (10 items):	✓	✓	✓
**Self-efficacy**	The Lewis´ Cancer self-efficacy scale [17] (validated by Northouse [5]) For **patients** (17 items) For **caregivers** (17 items)	✓	✓	✓
***Outcome measures used for the secondary endpoints***				
**Quality of life** (also covers additional secondary outcomes such as hopelessness, anxiety, depression, etc.)	For **patients** (23 items): - EORTC QLQ-C15-PAL [18] plustwo social functioning items (#26, 27) + one item about overall health (#29) from EORTC QLQ-C30 [19] - Social well-being scale from FACT-G [20] For **caregivers** (35 items): - The Caregiver Quality of Life Index-Cancer (CQOLC) [21]	✓	✓	✓
**Benefits of illness**	Benefits of illness scale [22] For **patients** (5 items) For **caregivers** (5 items)	✓	✓	✓
**Coping**	A shortened version of Brief Cope [23] (#1–3,5-10,13-16,19-21,23–26) For **patients** (20 items) For **caregivers** (20 items)	✓	✓	✓
**Dyad communication**	The five items ‘Active engagement scale’ from the ´Ways of giving support questionnaire´ [24]. Three scales (10 items) from the ‘Dyadic Coping Inventory’ [25]: ‘Stress communication by oneself’, ‘Stress communication by partner’ and ‘Evaluation of dyadic coping’. For **patients** (15 items) For **caregivers** (15 items)	✓	✓	✓
**Health economic measures**	EQ5D5L [26] and CSRI [27] For **patients** (23 items) For **caregivers** (14 items)	✓	✓	✓
***Background characteristics***				
**Socio demographics, illness-related factors, social factors**	A mix of socio-demographic items from different studies (self-constructed): - Sex, age, relationship status, living situation, having children, educational level, employment status, total monthly net income, financial difficulties related to physical condition or medical treatment, private medical insurance, religion, member of a minority ethnic group, dyad’s relationship For **patients** (14 items) For **caregivers** (15 items)	✓		
***Other aspects evaluated***				
**Items about computer skills**	Three FOCUS items about computer skills (self-constructed) For **patients** (2 items) For **caregivers** (2 items)	✓		
**Process evaluation**	FOCUS items asking about **experience** and **satisfaction** with the intervention. (self-constructed) For **patients** (12 items) For **caregivers** (12 items)		✓	
**Process evaluation**	**Experiences with the intervention**: (self-constructed) - Interviews with patients and family caregivers - Interviews with nurses delivering the intervention		✓	
	**Fidelity: FOCUS+** (self-constructed) Session characteristics (e.g. length, timing), random sample intervention checklists, random sample audio-taped intervention sessions		✓	
	**Fidelity: iFOCUS** (self-constructed) Data from web-based program (e.g. number of sessions logged into, time taken to complete session)		✓	
	**Routine data on recruitment** (self-constructed) (e.g. potential participants, initial engagement, eligible participants, enrolment)	✓		

^a^For T_1_, questionnaires can be filled in between T_0_ + 12 weeks minimum and T_0_ + 16 weeks maximum. For T_2_, questionnaires can be filled in between T_0_ + 24 weeks minimum and T_0_ + 28 weeks maximum

^b^All measures were validated in each of the participating countries

### Participant timeline

The face-to-face FOCUS+ intervention is a home-based intervention consisting of two 90-min home visits and one 30-min online video session, conducted by a trained intervention nurse who visits the dyads at home over 12 weeks, with 4 weeks between each session. The web-based iFOCUS intervention is a self-managed intervention that is completed autonomously by the patient-caregiver dyads at home. The iFOCUS intervention encompasses four sessions for the dyads (with 3 weeks between each session) over 12 weeks. Data will be collected three times from patient-caregiver dyads: 1) baseline measure (T_0_) after which the dyad will immediately be randomized to one of the study arms, 2) first follow-up at 12 weeks after the baseline (T_1_), and 3) second follow-up at 24 weeks after baseline (T_2_). Figure 2 provides a flowchart of the participant timeline and the data collection.

### Sample size calculation

We consider the demonstration of an intervention effect (for each of both interventions) on at least one of the primary endpoints for either the patient or the caregiver as a success. A pre-determined strict fixed sequence (FS) procedure defines the prospectively hierarchical ordering of the endpoints, for this study the hierarchical order is emotional functioning (1) and self-efficacy (2). Testing of null hypotheses proceeds according to their hierarchical order, that is, H(1)0 is tested first at a significance level of 5%, and if H*(*1*)*0 is rejected then H*(*2*)*0 is tested at the same significance level, otherwise H*(*2*)*0 is not tested at all. The strict FS approach has the highest power for testing the first hypothesis (outcome: emotional functioning) compared to the other methods, as it does not save any portion of alpha for testing later hypotheses [10]. The reference mean value for emotional functioning from EORTC for all cancer patients, stage III-IV is 71.5 (SD: 23.8) [28]. Alpha is set at 0.0125 instead of 0.05 to account for multiplicity (2 comparisons with control group * 2 participant groups [patients and caregivers]). We set 1-beta (i.e. statistical power) at 0.9. The expected difference between the control group and the intervention arms in the primary outcomes is 0.375 SD at T1 (12 weeks).

With these parameters *n* = 203 is needed in each arm across all countries (i.e. 609 in total). Anticipating a 65% retention rate at T1, which is more conservative than found in previous studies in the USA on the FOCUS interventions [5–7] due to the advanced cancer population included in this study, 938 dyads must be enrolled across the 6 countries (313 per group). This means that 156 dyads (i.e. 52 in each of the 3 arms) need to be enrolled in each country. Based on previous studies in the US [5–7] we expect an enrolment rate of 55% of those dyads referred to the study, meaning that about 282 dyads will need to be screened and identified in each country. The feasibility of recruitment has been evaluated based on previous research and discussions with clinicians in eligible hospitals.

### Recruitment

Staff in each department in the participating hospitals will screen for patients meeting the eligibility criteria. After eligible patients are identified, the study is orally presented to the patient-caregiver dyad and they are invited to participate in the study by a research assistant. This research assistant will explain the study and refer the patient and their family caregiver to the contact person of the research team. A data collector will make an appointment with the dyad at home (or preferred location of the dyad) to obtain informed consent.

The recruitment period will last a total of 12 months. Screening will continue until the target population is achieved. Based on conservative estimations of the number of eligible dyads that could be enrolled, obtaining the numbers required by the power calculations is realistic.

## Assignment of interventions

### Allocation

Randomization will be performed per a computer randomization schedule with a 1:1:1 allocation ratio to one of the two intervention arms or the standard care arm (control group) using simple randomization. Participants will be randomized using the Mersenne Twister Random Number Generator, which is an online, central randomization service [29]. Each dyad will be randomized to one of the three study arms. Recruitments for the study within a country will continue until at least 52 dyads were randomized.

Allocation concealment will be ensured, as the randomization service will not release the randomization code until the patient has been recruited into the trial, which takes place after all baseline measurements have been completed.

All dyads who fulfil the inclusion criteria, who give consent for participation, and complete the baseline measurements (obtained by the data collector) will be randomized. Randomization will be immediately requested after completion of the baseline questionnaires from the online service Mersenne Twister Random Number Generator by the data collector, who is then able to inform the dyads of their allocation to one of the study arms. Randomization will thus be conducted without any influence of the principal investigators, raters, or clinicians. Research staff responsible for the data analyses of the trial will not be allowed to receive any information about the group allocation.

### Blinding

Due to the nature of the intervention neither the patient-caregiver dyads nor the intervention nurses (for the face-to-face FOCUS+ intervention) can be blinded to allocation. Those conducting the data analyses will remain blind as to what trial arm dyads were randomized to until the end of the last data-collection point.

## Data collection, management, and analysis

### Data collection methods

**Fig. 3:**
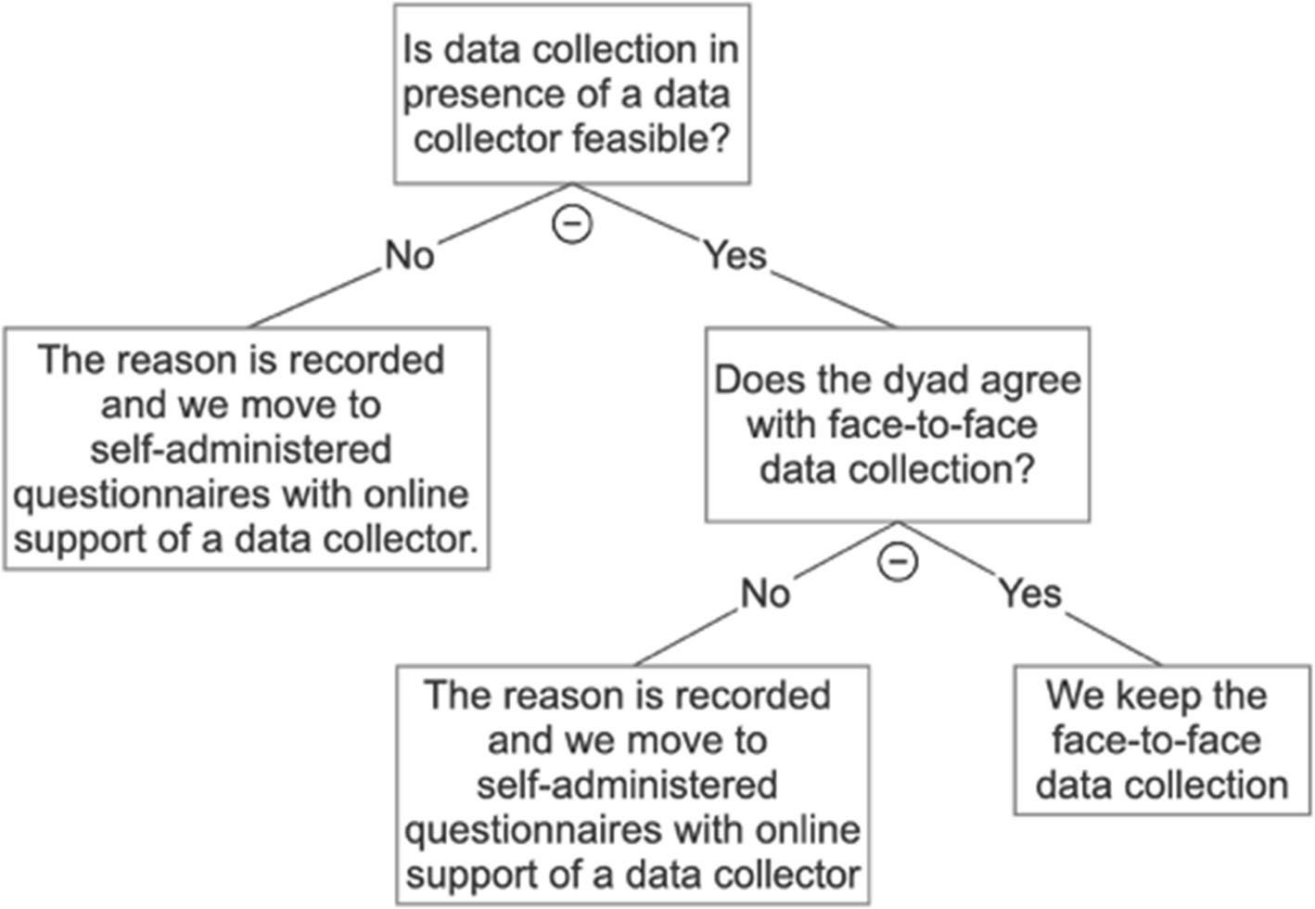
Decision-making process for data collection procedure in light of possible COVID-19 developments

The outcomes and outcome measures are described in 2.4. Quantitative data will be collected via computer-assisted self-interview in the presence of a data collector that can assist dyads with the completion of the questionnaires at T_0_ and T_1_. In case of tightened measures to contain the COVID-19 pandemic, data collection at T_0_ and T_1_ via a GDPR-approved online platform (e.g. MS Teams, Zoom,..) will be allowed, if necessary. The decision-making process is visualized in Fig. 3 and will be recorded for each dyad in each country. At T_2_, questionnaires will be self-administered online (without the presence and support of a data collector), with a telephone follow-up in case of nonresponse. The questionnaires were pilot tested among a small number of dyads before the start of the study to identify problems and limitations concerning respondent comprehension and acceptability.

To address the aim regarding the evaluation of the process of implementation both quantitative data and qualitative data will be collected throughout and after the interventions in each country and used for the evaluation:

1. Detailed documentation of the recruitment process allows us to obtain information about the way patients and family caregivers were recruited and their reasons for participating or not.
2. To measure the extent to which the intervention is implemented as intended (fidelity and consistency across the countries), the nurses delivering the intervention will be required to audio record intervention sessions, allowing members of the research team to complete a structured intervention checklist on a random sample of recordings every 3 months to record any deviations from the program. Adherence to the intervention is monitored by the use of these checklists.
3. Repeated interviews with nurses who delivered the face-to-face FOCUS+ intervention in each country (*n* = 6 in total) and research staff supporting the delivery of the Web intervention (*n* = 6 in total) are also planned. These interviews will provide information on perceived barriers and facilitators to the delivery of the intervention and their experience or observations on the elements of the interventions that may represent mechanisms/agents of change. If necessary, in light of the COVID-19 pandemic, these interviews will take place online.
4. A selection of 10 dyads who received either of the interventions is interviewed in every country to explore their perception of the intervention, their experience of the impact of the program, the core elements of the program that were central to their experience, and any recommendations for the further development of the intervention. The interviews will be conducted with a minimum of 10 dyads in each country, to include four dyads who complete either intervention and two dyads who did not complete either intervention. This information will allow consideration of the mechanism of impact or agents of change. All interviews will be audio-recorded and transcribed verbatim in the local language.

To reduce loss to follow-up and improve retention, retention gifts with the logo of the study on them will be provided after the completion of each data collection. These gifts are a coffee mug at T_0_, a magnetic notepad at T_1_, and plant seeds at T_2_. With these gifts, we intend to encourage participants to complete the data collections.

### Data management

The data collected through the questionnaires will be coded and stored in a safe data environment. Data cleaning will be carried out following a data cleaning protocol already developed and used by the coordinating partner Vrije Universiteit Brussel.

The consortium certifies that all research activities will adhere most strictly to all applicable legal, ethical, and safety provisions of the individual states and the EU. Participants will conform to relevant EU legislation including (1) The Charter of Fundamental Rights of the EU, December 2009 and (2) EU Regulation 2016/679 on the protection of natural persons concerning the processing of personal data and on the free movement of such data (GDPR).

The overall trial manager (Vrije Universiteit Brussel) will take the overall responsibility for data management throughout the DIAdIC project. Ultimately responsibilities for data management will be transferred to consortium partners to allow a collaborative and efficient collection of research findings throughout the time of the project. The Data Management Plan is added as a supplemental file.

### Statistical methods/data analysis

#### Quantitative analysis

Four main analyses will take place.

1. Primary hypotheses testing
  - *Testing the null hypothesis of the first primary endpoint: emotional functioning.*

The effectiveness of the FOCUS+ face-to-face intervention and the iFOCUS web-based intervention will be compared with the standard care (control group) for each participant population (patients/caregivers) separately. In total, 4 comparisons are performed for one outcome variable (alpha = 0.0125). The hypotheses related to the first primary outcome (emotional functioning) will be tested using a mixed model (per participant population) with the T1 measurement value for emotional functioning as the outcome variable, recruitment center as random effect and randomization group, and the baseline measure of emotional functioning (T0) as predictor variables. We will perform analyses on both ‘intention-to-treat’ and per-protocol principles. The primary principle is intention-to-treat. After completion of the baseline measurement (T0), dyads will be randomized to one of the trial arms. All randomized dyads will be included in the mixed model. Multiple imputations will be applied. Predictors for the imputation model will include the baseline measurement, randomization group, age, and other variables (e.g. severity of the illness). The secondary principle is the per-protocol analysis that functions as a sensitivity analysis. The per-protocol population will be defined as dyads who have completed all sessions of the FOCUS+ or iFOCUS intervention (except for dyads in the control group) and T1 measurement. By including the baseline measurement as a predictor variable (ANCOVA), preexisting differences will be controlled for, enhancing the sensitivity of the analyses. To interpret the magnitude of the effects for the different outcomes, we will estimate effect sizes (Cohen’s d).

b. *Testing the null hypothesis of the second primary endpoint: self-efficacy (the Lewis´ Cancer self-efficacy scale from FOCUS)*

As per the fixed sequence (FS) procedure, the null hypotheses of the second primary endpoint (self-efficacy) will only be tested if a significant result is found for the first primary endpoint (emotional functioning). The same strategy is then followed for the analyses as for the first primary endpoint, with an alpha level of 0.0125.

(2) Secondary hypotheses testing

All identified secondary endpoints (Quality of Life [including separate items of hopelessness, anxiety, depression], benefits of illness, coping, dyad communication, all at T1) will be evaluated by testing the FOCUS+ and iFOCUS against care as usual (control group) for each participant population (patients/caregivers) separately. In total, 4 comparisons are performed for each outcome variable. For each secondary outcome variable, a mixed model is applied with the T1 measurement value as the outcome variable, recruitment center as random effect, and randomization group and baseline measurement of the variable (T0) as predictor variables. We will perform analyses on both ‘intention-to-treat’ and per-protocol principles, applying the same principles as described above.

By including the baseline measurement as a predictor variable (ANCOVA), preexisting differences will be controlled, enhancing the sensitivity of the analyses. To interpret the magnitude of the effects for the different outcomes, we will estimate effect sizes (Cohen’s d). All statistical tests will be two-sided and considered significant if *p* < 0.0125.

All primary outcomes and secondary outcomes as listed above will also be analysed at T2 (6 months) to evaluate longer-term effects, using the same analysis procedures.

The cost-effectiveness of the interventions will be determined by analyzing patterns and costs of healthcare utilization and effects on quality of life. Costs will be estimated by combining the reported frequency of health care use by participants with country-specific unit costs for each service domain. Quality of life will be measured by combining EQ5D5L with country-specific preference weights. We will evaluate the robustness and consistency of results in population-specific outcome measures EORTC, FACT-G and CQOLC. Data will also be collected on the types and amounts of informal care provided to patients in each arm of the study, to investigate if the amount or patterns of informal care change as a result of the intervention. The outputs will be mean costs of care for patients in each arm of the study, cost per year of life gained (if survival is affected significantly by the intervention and the costs in the intervention groups overall are higher), and (if appropriate) the additional costs of achieving a better quality of life outcomes (including estimates of cost per quality-adjusted life-year gained).

(3) Exploratory hypotheses testing

For all exploratory endpoints, two-sided statistical tests will be considered significant if *p* < 0.05.

- For the outcomes that are measured identically for the patient and the caregiver, we will assess the effect on the dyad as a whole (i.e. both patient and family caregiver). For the outcome instruments that led to comparable estimated differences between FOCUS+ and standard care and iFOCUS and standard care, the effect will be assessed on the dyad as a whole by adding an extra level (dyad) to the linear regression model.
- For each of the primary and secondary endpoints, subgroup analyses will be performed using formal interaction tests to explore the extent to which the outcomes of the trial differ by country, gender, and socioeconomic status. Interaction terms between respectively country, gender, and socioeconomic status on the one hand and the trial arms on the other hand will be added to the analysis models. For the country variation multilevel mixed model analyses will also be performed to additionally account for potential clustering by country (i.e. participants nested within a country). Outcomes will be analysed with country as random factor.

(4) Other analyses
  - Background reports describing care as usual for people with advanced cancer will facilitate the understanding of the results of the between-country comparisons.
  - Process evaluation of the implementation of the interventions will be analyzed following the MRC framework for evaluating complex interventions [30], integrating normalization process theory (NPT) [31] and the RE-AIM framework [32]. Data analysis for the process evaluation will include a) standard statistical descriptions of the quantitative data from the intervention checklist and routine monitoring to describe adherence to the implementation. This analysis will determine cut-off points for good intervention adherence and, hence, inform the per-protocol analyses; b) analyses of the qualitative data (semi-structured interviews with patients and their family caregiver and post-intervention interviews with the nurses who delivered the face-to-face FOCUS+ intervention) will be performed (see below - Qualitative analysis).

#### Qualitative analysis

With the transcription of interviews into the local language, the analysis process will involve a collaborative process (Richards & Hemphill, 2017) [13] involving researchers from each partner site collecting data. The general process will be guided by the thematic analysis framework proposed by Braun and Clarke (2006) [28], with quality ensured with criteria for trustworthiness reported by Nowell et al. (2017) [29] and applied procedures for auditing the analysis process. Thematic analysis allows for both inductive and deductive analysis and can be implemented with a range of computer-based software to support the management of the analysis process (e.g., NVIVO, MAXQDA).

Deductive analysis will be informed by semantic information sought from the interview (i.e., were participants satisfied, were particular elements of the programmes described as positive or negative) and themes evident in previous evaluations of the Focus Intervention. This will involve developing themes in advance of the analysis process and assessing the presence or absence of these themes across the data. Inductive analysis will be structured using the objectives of the process evaluation to target key topics, with more latent or interpretative themes isolating more experiential findings from the data. Qualitative analysis will be conducted at two levels, an initial assessment of themes in each data source (stakeholders, staff, researchers, patients and carers, different language groups) followed by a higher-level analysis of superordinate themes of convergence and divergence evident across groups. Additional strategies for managing the potential impact of multilingual analysis are recommended by Richards and Hemphill (2017) [13], including peer debriefing during the process of coding and the development of candidate themes, triangulation across researchers and language sources. Analysis will be informed by an open discussion of conceptual issues in the data, as highlighted in Larkin, Dierckx de Casterlé, and Schotsmans (2007) [30], to explore variations in interpretation and identify shared meaning relevant to the focus of the process evaluation.

## Data monitoring

### Data monitoring

An Ethical and Data Monitoring Board will monitor the intervention and data collection and any adverse events will be reported to the Board. This Board consists of an Ethics Evaluator and the independent trial monitors. The Ethical and Data Monitoring Board will review the accumulating data periodically and determine if the trial should be modified or discontinued. The monitoring visits will be conducted by national independent trial monitors.

### Harms

The FOCUS+ and iFOCUS interventions are non-invasive psycho-educational interventions focused on the provision of information; they are not intended to be a therapeutic or cognitive behavioral treatment. Based on previous research with similar FOCUS interventions in the USA, it is unlikely that there will be any adverse effects. However, a protocol will be in place for trial managers, data collectors, and intervention nurses involved in delivering the intervention on how to handle unanticipated adverse events should they occur.

### Auditing

Auditing will be independent of investigators and the sponsor. The first Monitoring Visit following initiation of each site will take place approximately 6 weeks after the inclusion of the first dyad, which allows that the first dyads are enrolled in the study and have completed already some parts of the face-to-face FOCUS+ intervention or web-based iFOCUS intervention to be monitored. Subsequent monitoring visits will be conducted in weeks 22, 38, 54 and 70 of the trial. A close-out monitoring visit will be planned in week 73. The interval for Monitoring Visits may be longer or shorter than stated above, depending on enrolment rate, site compliance, or quality issues.

## Ethics and dissemination

### Research ethics approval

The protocol is already approved by Commissie Medische Ethiek UZ Gent, Belgium, 22/10/2020; Ethisch Comité AZ Maria Middelares, Belgium, 16/11/2020; St. Vincent’s University Hospital Research Ethics Committee, Ireland, 04/12/2020; Comitato Etico dell’Area Vasta Emilia Nord (AVEN), Italy, 13/04/2021; NHS/HSC Research Ethics Committee, United Kingdom, 05/03/2021; Medisch Ethische Toetsings Commissie Erasmus Medisch Centrum Rotterdam, the Netherlands, 15/01/2021. The Denmark Scientific Ethical Committee system (protocol no. 19043825) determined that the protocol did not require further formal approval on 22/08/2019; this decision was confirmed after the submission of the final protocol.

### Protocol amendments

Any modifications to the protocol which may impact the conduct of the study, the potential benefit of participants, or may affect patient safety, including changes of study objectives, study design, patient population, sample sizes, study procedures, or significant administrative aspects will require a formal amendment to the protocol. Such amendment will be agreed upon by the DIAdIC consortium partners and approved by the Ethics Committee before implementation.

### Consent

The data collector will obtain informed consent from dyads willing to participate. Patients and caregivers will be given ample time to consider participation and they will be assured that they are free to withdraw from participating in the study without any effect on their care. Participants will be made aware that consent is fluid and that they have the right to withdraw their consent at any time throughout the study without any negative impact on their healthcare or management. Written consent will be obtained without any coercion of study participants. The research team will provide all participants with full disclosure about the nature and goal of the study. Participants will be given the opportunity to ask questions before they decide if they want to participate.

### Confidentiality

All project partners will take all required steps to guarantee compliance with the provisions of the EU Regulation 2016/679 on the protection of natural persons concerning the processing of personal data and on the free movement of such data (GDPR) as well as the related legislation of the Member States of the project partners.

### Declaration of interests

There are no financial and other competing interests for principal investigators for the overall trial and each study site.

### Access to data

All data collected will be stored on a dedicated server in France which has accredited medical data hosts. Data access is strictly limited to those who require access to perform the study. The people who have access will be appointed by the data protection officer of the coordinating partner (VUB).

### Ancillary and post-trial care

Dyads in the trial are referred to specific existing services or community resources based on the problems or needs identified during the intervention sessions (ancillary care) or after the trial (post-trial care).

### Dissemination policy

The DIAdIC project will use an extensive dissemination and exploitation strategy and apply a broad range of communication activities to inform the scientific community about the project results and their implications. There are six dissemination objectives; (1) at least 6 Ph.D. dissertations, (2) publication in open access, green and gold international journals, (3) publication in national topic-specific journals, (4) communication to and interaction with all scientific stakeholders through active contributions at international conferences, (5) events and workshops organized at the premises of the DIAdIC partners and (6) an end-of-project conference.

## Discussion

This project aims to study the effect of two psycho-educational interventions on emotional functioning and self-efficacy of patients with advanced cancer and their informal caregivers. DIAdIC is a large-scale study conducted across different countries and provides knowledge about intervention dosage and different modes of administration. Psychosocial support and education for people with advanced cancer and their family caregivers can substantially reduce their distress, improve their QoL, and prevent avoidable health resource use [4, 8]. Often, family caregivers have a double role: as a provider of care to the person with cancer, but also as a person in need of support for themselves [33]. Both interventions focus on the empowerment of non-professionals (the family caregiver and the patient with advanced cancer) by improving their mutual communication, assisting them to identify positive aspects related to their situation, and increasing their self-efficacy.

Few studies have systematically assessed the cost-effectiveness of large scale psychosocial and psychoeducational interventions. As the cost-effectiveness of the interventions will be determined by analyzing patterns and costs of healthcare utilization and effects on QoL, the results of this RCT can thus contribute to the search for cost-effective novel interventions that can relieve constraints on professional healthcare.

The cross-country setup in six European countries allows for a comparison of the effectiveness of the interventions in different healthcare systems and regimes across Europe. The DIAdIC interventions are complex interventions where contextual factors such as country-specific healthcare systems and dyad characteristics influence the mechanisms of action of the interventions. Therefore, a thorough process evaluation following the normalization process theory and the RE-AIM framework is embedded in the study. This analysis will determine cut-off points for good intervention adherence and, hence, inform the per-protocol analyses.

We anticipate some limitations and challenges in the DIAdIC trial. First, the inclusion of participants might be difficult for several reasons. As the target population includes patients with advanced cancer with a limited life expectancy of maximum 2 years and their family caregivers, eligible patient-caregiver dyads might be reluctant to enroll in a time-consuming trial. They might feel too tired or want to spend the time they have left doing different things. Additionally, a dyad can only participate in the trial when both the family caregiver and the patient agree to participate. Hence, we need consent from two people to include one participating entity or dyad in the study, which can cause the enrolment rate to be lower than other studies. Second, to address all the different outcomes of the DIAdIC trial, data will be collected three times from patient-caregiver dyads through long self-administered questionnaires, which may cause a considerable burden and can lead to high nonresponse. To limit the risk of nonresponse, the data collector is present at T_0_ and T_1_. For T_2_ the data collector will do a telephone follow-up in case of nonresponse. Third, the complexity of the study and the many aspects of tailoring and context does not allow for unambiguous identification of the active component of the interventions. This challenge is anticipated by conducting a thorough process evaluation to determine the mechanisms of action of the interventions. Fourth, as a consequence of the COVID-19 pandemic, the mode of delivery of the FOCUS+ intervention can differ between dyads, i.e. either face-to-face or via an online video platform. By recording the mode of delivery of the FOCUS+ intervention for each dyad in each country, within-group variations in effectiveness can be monitored.

## Supplementary Information

**Additional file 1.**

## Data Availability

Sharing of datasets will be defined on a case-by-case basis. Based on the type of dataset, it will be decided which datasets can be made public, shared or have to be kept confidential. However, we will publicly share certain research data under ‘open access’ on the project repository, as mandated by the EU: - Public-use files: Datasets not containing personal data, and the content of the web-based tool or any other tools that will be developed in the project, will be made publicly available and will be published online (e.g. in an open access repository such as https://zenodo.org/). After removing direct and indirect identifiers, data can also be shared directly via the DIAdIC website to allow an easy access for any researcher or civilian who is interested. - Restricted-use data files: files containing confidential information and/or direct and indirect identifiers that cannot be shared publicly. These files will be under ‘restricted access’ on the repository. Datasets containing personal data will be made available upon request by mail and upon signing a data user agreement, to prevent further dissemination of personal data beyond the researchers that get approval for re-use. Research data from DIAdIC will be supplied to an online data repository as soon as publishable and within the duration of the project.

## Abbreviations

DIAdIC: Diadic Interventions for people with Advanced and their Informal Caregiver
QoL: Quality of Life
RCT: Randomized Controlled Trial
SPIRIT: Standard Protocol Items: Recommendations for Interventional Trials
GDPR: General Data Protection Regulation
