# Supplementary material for: Effectiveness of a nurse-delivered (FOCUS+) and a web-based (iFOCUS) psychoeducational intervention for people with advanced cancer and their family caregivers (DIAdIC): study protocol for an international randomized controlled trial
Source: BMC Palliat Care. 2021 Dec 28;20:193. doi: 10.1186/s12904-021-00895-z (PMC8713043; doi:10.1186/s12904-021-00895-z)
Supplement: Supplementary file 1 — Additional file 1. [file 12904_2021_895_MOESM1_ESM.docx]

**
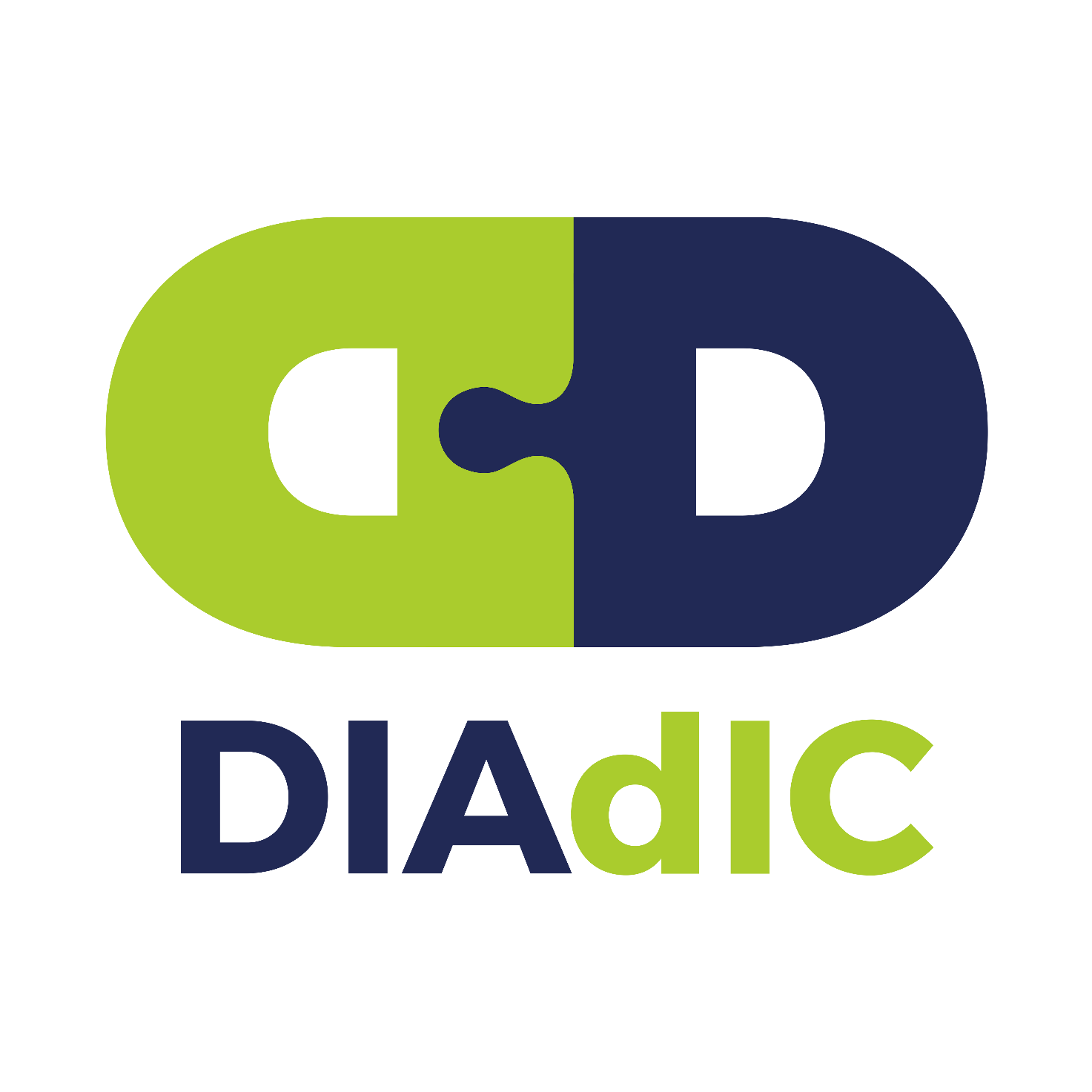
**

**Questionnaire for patients**

**Self-made process evaluation items (additional to T_1_)**

We are very interested in your evaluation of the FOCUS/iFOCUS programme. Please select answers to the questions below that best describe your thoughts and feelings about the programme.

| For each of the following questions about your overall experience using the programme, choose a number from 1 to 7 that best represents your view. To indicate your view, mark the number. | | | | | | | | |
| --- | --- | --- | --- | --- | --- | --- | --- | --- |
| 1. How useful was the programme to meeting your needs? | | | | | | | | |
|  | 1 | 2 | 3 | 4 | 5 | 6 | 7 |  |
|  | Not at all useful |  |  |  |  |  | Very useful |  |
| 1. To what extent were you satisfied with the programme? | | | | | | | | |
|  | 1 | 2 | 3 | 4 | 5 | 6 | 7 |  |
|  | Very dissatisfied |  |  |  |  |  | Very satisfied |  |
| 1. To what extent are you interested in using the progamme materials for support in the future? | | | | | | | | |
|  | 1 | 2 | 3 | 4 | 5 | 6 | 7 |  |
|  | Not at all interested |  |  |  |  |  | Very interested |  |

| For each of the following questions about your overall experience using the programme, choose the response option/answer that best represents your view. To indicate your view, mark the answer. | | | | | | | | | | | | | | | |  |
| --- | --- | --- | --- | --- | --- | --- | --- | --- | --- | --- | --- | --- | --- | --- | --- | --- |
| 1. To what extent did the programme meet your expectations? | | | Fell below my expectations | | | | Met my expectations | | | | | Exceeded my expectations | | | |  |
| 1. How would you rate the amount of information provided by the programme? | | | Not enough information | | | | Just the right amount | | | | | Too much information | | | |  |
| 1. How often did you use the core materials provided as part of the programme? | | | Never | | Only once/twice overall | | | | Monthly | | Weekly | | | Daily | |  |
| For the following question about your overall experience using the programme, choose the response option/answer that best represents your view. To indicate your view, mark the answer. | | | | | | | | | | | | | | | |  |
| 1. Is the programme something that you would recommend to other people coping with cancer? | | | No | | | | Don’t know | | | | | Yes | | | |  |
| Using the following scale (1 = “strongly disagree,” 7 = “strongly agree”), please rate how much you agree or disagree with these statements. To indicate your view, mark the number. | | | | | | | | | | | | | | | | |
| 1. The information I received in the programme was important to me. | | | | | | | | | | | | | | | | |
|  | 1 | 2 | | 3 | | 4 | | 5 | | 6 | | | 7 | |  | |
|  | Strongly Disagree |  | |  | |  | |  | |  | | | Strongly Agree | |  | |
| 1. The information in the programme made me feel uncomfortable. | | | | | | | | | | | | | | | | |
|  | 1 | 2 | | 3 | | 4 | | 5 | | 6 | | | 7 | |  | |
|  | Strongly Disagree |  | |  | |  | |  | |  | | | Strongly Agree | |  | |
| 1. The programme took too much time. | | | | | | | | | | | | | | | | |
|  | 1 | 2 | | 3 | | 4 | | 5 | | 6 | | | 7 | |  | |
|  | Strongly Disagree |  | |  | |  | |  | |  | | | Strongly Agree | |  | |
| 1. I thought the programme was easy to understand. | | | | | | | | | | | | | | | | |
|  | 1 | 2 | | 3 | | 4 | | 5 | | 6 | | | 7 | |  | |
|  | Strongly Disagree |  | |  | |  | |  | |  | | | Strongly Agree | |  | |
| 1. I can use the information from the programme in my day-to-day life. | | | | | | | | | | | | | | | | |
|  | 1 | 2 | | 3 | | 4 | | 5 | | 6 | | | 7 | |  | |
|  | Strongly Disagree |  | |  | |  | |  | |  | | | Strongly Agree | |  | |

**Socio-demographic items at T_0_**

The final questions ask you to provide some important background information about you and your current situation. Please tick the box that best applies.

| 1. Are you:   ❑ Male  ❑ Female  ❑ Other  ❑ Prefer not to answer |  | | | |
| --- | --- | --- | --- | --- |
| 1. How old are you?   _________ years |  | | | |
| 1. What is your current relationship status? | |  | | |
| ❑ Married or in a relationship  ❑ Separated/divorced/widowed  ❑ Single/not in a relationship  ❑ Prefer not to answer   1. Do you have children? (tick all the boxes that apply)   ❑ I have children under the age of 18  ❑ I have adult children age 18 years or older  ❑ I don´t have any children | | |  | |
| 1. Do you live:   ❑ In a private household (flat, house etc.)  ❑ In an institution/care facility (nursing home, sheltered housing etc.)  ❑ Other. Please specify:________________ | | | |  |
| 1. Together with whom do you live? (tick all the boxes that apply)   ❑ With a spouse/partner  ❑ With children under the age of 18  ❑ With adult children age 18 years or older  ❑ With other persons, please specify:________________  ❑ I live alone   1. What is your relationship to the person who is participating in this study with you?   I am his/her…  ❑ Spouse/partner  ❑ Parent  ❑ Sister/brother    ❑ Daughter/son  ❑ Other relative  ❑ Friend    ❑ Other, please specify: _______________________ | | | |  |
| 1. What is the highest level of education you have completed?   ❑ Pre-primary education  ❑ Primary school education  ❑ CSE, ‘O’ levels, GCSEs, entry level foundation diploma  ❑‘A’ levels, AS levels, VCEs Higher diploma  ❑ NVQ level 2, Intermediate GNVQ, City and Guilds, BTEC first general diploma, RSA diploma, Apprenticeship  ❑ NVQ level 4, HND Foundation Degree, Diploma of Higher Education  ❑ Bachelor’s degree, Graduate Certificate, Graduate Diploma, Professional Graduate certificate in education  ❑ MSc, NVQ level 5, Postgraduate Diploma, Postgraduate certificate  ❑ PhD, Dphil, Professional doctorate  ❑ Prefer not to answer | | | |  |
| 1. Are you currently employed? Tick one box only.   ❑ Yes, but I am currently on sick / care leave (full time or part time)  ❑ Yes, I work full time  ❑ Yes, I work part time. Number of hours: __________ hours  ❑ No, I am unemployed  ❑ No, I am a homemaker  ❑ No, I am retired    ❑ No, I am a student  ❑ Other, please specify: __________________________  ❑ Prefer not to answer   1. What is the total monthly net income of your household (i.e your income after tax and other deductions)?   ❑ Approximately £0-894 per month  ❑ Approximately £895-1789 per month  ❑ Approximately £1790-2684 per month  ❑ Approximately £2685-3579 per month  ❑ Approximately £3580-4473 per month  ❑ Approximately £4474-5368 per month  ❑ More than £5369 per month  ❑ Don´t know  ❑ Prefer not to answer   1. Which of the following descriptions comes closest to how you feel about your household income nowadays?   ❑ Living comfortably on present income  ❑ Coping on present income  ❑ Difficult on present income  ❑ Very difficult on present income  ❑ Don´t know  ❑ Prefer not to answer   1. Has your physical condition or medical treatment caused you financial difficulties?   ❑ Not at all  ❑ A little  ❑ Quite a bit  ❑ Very much  ❑ Prefer not to answer  The following question concerns an entirely different topic.   1. Do you agree with the following statement: ´I believe in God or in someone or something greater than myself´.   ❑ Not at all  ❑ A little  ❑ Quite a bit  ❑ Very much    ❑ Prefer not to answer | | | |  |
| 1. Your country of birth: ___________   If your mother or father was born in another country, please state it below:    Country of birth of mother: _______________  Country of birth of father: _______________ | | | |  |

**
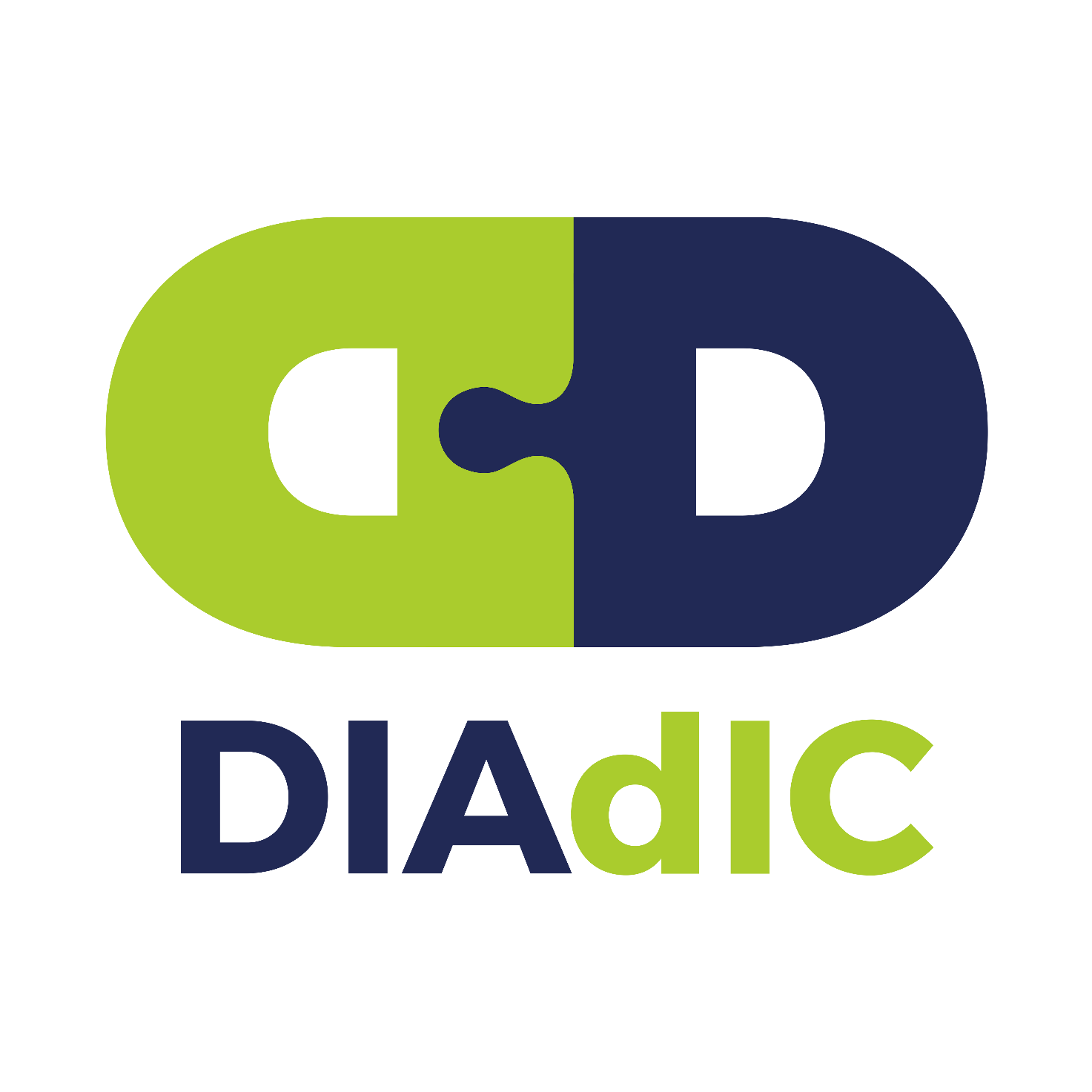
**

**Questionnaire for carers**

**Self-made process evaluation items (additional to T_1_)**

We are very interested in your evaluation of the FOCUS/iFOCUS programme. Please select answers to the questions below that best describe your thoughts and feelings about the programme.

| For each of the following questions about your overall experience using the programme, choose a number from 1 to 7 that best represents your view. To indicate your view, mark the number. | | | | | | | | |
| --- | --- | --- | --- | --- | --- | --- | --- | --- |
| 1. How useful was the programme to meeting your needs? | | | | | | | | |
|  | 1 | 2 | 3 | 4 | 5 | 6 | 7 |  |
|  | Not at all useful |  |  |  |  |  | Very useful |  |
| 1. To what extent were you satisfied with the programme? | | | | | | | | |
|  | 1 | 2 | 3 | 4 | 5 | 6 | 7 |  |
|  | Very dissatisfied |  |  |  |  |  | Very satisfied |  |
| 1. To what extent are you interested in using the progamme materials for support in the future? | | | | | | | | |
|  | 1 | 2 | 3 | 4 | 5 | 6 | 7 |  |
|  | Not at all interested |  |  |  |  |  | Very interested |  |

| For each of the following questions about your overall experience using the programme, choose the response option/answer that best represents your view. To indicate your view, mark the answer. | | | | | | | | | |  |
| --- | --- | --- | --- | --- | --- | --- | --- | --- | --- | --- |
| 1. To what extent did the programme meet your expectations? | Fell below my expectations | | | | Met my expectations | | | Exceeded my expectations | |  |
| 1. How would you rate the amount of information provided by the programme? | Not enough information | | | | Just the right amount | | | Too much information | |  |
| 1. How often did you use the core materials provided as part of the programme? | Never | | Only once/twice overall | | | Monthly | | Weekly | Daily | |
| 1. Is the programme something that you would recommend to other people coping with cancer? | | No | | Don’t know | | | Yes | | |  |

| Using the following scale (1 = “strongly disagree,” 7 = “strongly agree”), please rate how much you agree or disagree with these statements. To indicate your view, mark the number*.* | | | | | | | | |
| --- | --- | --- | --- | --- | --- | --- | --- | --- |
| 1. The information I received in the programme was important to me. | | | | | | | | |
|  | 1 | 2 | 3 | 4 | 5 | 6 | 7 |  |
|  | Strongly Disagree |  |  |  |  |  | Strongly Agree |  |
| 1. The information in the programme made me feel uncomfortable. | | | | | | | | |
|  | 1 | 2 | 3 | 4 | 5 | 6 | 7 |  |
|  | Strongly Disagree |  |  |  |  |  | Strongly Agree |  |
| 1. The programme took too much time. | | | | | | | | |
|  | 1 | 2 | 3 | 4 | 5 | 6 | 7 |  |
|  | Strongly Disagree |  |  |  |  |  | Strongly Agree |  |
|  | | | | | | | | |
| 1. I thought the programme was easy to understand. | | | | | | | | |
|  | 1 | 2 | 3 | 4 | 5 | 6 | 7 |  |
|  | Strongly Disagree |  |  |  |  |  | Strongly Agree |  |
| 1. I can use the information from the programme in my day-to-day life. | | | | | | | | |
|  | 1 | 2 | 3 | 4 | 5 | 6 | 7 |  |
|  | Strongly Disagree |  |  |  |  |  | Strongly Agree |  |

**Socio-demographic items at T_0_**

The final questions ask you to provide some important background information about you and your current situation as a carer to a person with cancer. Please tick the box that best applies.

| 1. Are you:   ❑ Male  ❑ Female  ❑ Other  ❑ Prefer not to answer |  | | | | |
| --- | --- | --- | --- | --- | --- |
| 1. How old are you?   _________ years |  | | | | |
| 1. What is your current relationship status? | |  | | | |
| ❑ Married or in a relationship  ❑ Separated/divorced/widowed  ❑ Single/not in a relationship  ❑ Prefer not to answer |  | | | | |
| 1. Do you have children? (tick all the boxes that apply)   ❑ I have children under the age of 18  ❑ I have adult children age 18 years or older  ❑ I don´t have any children | | |  | | |
| 1. Do you live:   ❑ In a private household (flat, house etc.)  ❑ In an institution/care facility (nursing home, sheltered housing)  ❑ Other. Please specify:________________ | | | | |  |
| 1. Together with whom do you live? (tick all the boxes that apply)   ❑ With a spouse/partner  ❑ With children under the age of 18  ❑ With adult children age 18 years or older  ❑ With other persons, please specify:________________  ❑ I live alone   1. Do you live together with the person with whom you participate in this study?   ❑ Yes  ❑ No   1. What is your relationship to the person who is participating in this study with you?   I am his/her…  ❑ Spouse/partner  ❑ Parent  ❑ Sister/brother    ❑ Daughter/son  ❑ Other relative  ❑ Friend  ❑ Other, please specify: _______________________ | | | | |  |
| 1. What is the highest level of education you have completed?   ❑ Pre-primary education  ❑ Primary school education  ❑ CSE, ‘O’ levels, GCSEs, entry level foundation diploma  ❑‘A’ levels, AS levels, VCEs Higher diploma  ❑ NVQ level 2, Intermediate GNVQ, City and Guilds, BTEC first general diploma, RSA diploma, Apprenticeship*)*  ❑ NVQ level 4, HND Foundation Degree, Diploma of Higher Education  ❑ Bachelor’s degree, Graduate Certificate, Graduate Diploma, Professional Graduate certificate in education  ❑ MSc, NVQ level 5, Postgraduate Diploma, Postgraduate certificate  ❑ PhD, Dphil, Professional doctorate  ❑ Prefer not to answer | | | | |  |
| 1. Are you currently employed? Tick one box only.   ❑ Yes, but I am currently on sick / care leave (full time or part time)  ❑ Yes, I work full time  ❑ Yes, I work part time. Number of hours: __________ hours  ❑ No, I am unemployed  ❑ No, I am a homemaker  ❑ No, I am retired    ❑ No, I am a student  ❑ Other, please specify: __________________________  ❑ Prefer not to answer   \| 1. What is the total monthly net income of your household (i.e your income after tax and other deductions)?   ❑ Approximately £0-894 per month  ❑ Approximately £895-1789 per month  ❑ Approximately £1790-2684 per month  ❑ Approximately £2685-3579 per month  ❑ Approximately £3580-4473 per month  ❑ Approximately £4474-5368 per month  ❑ More than £5369 per month  ❑ Don´t know  ❑ Prefer not to answer   1. Which of the following descriptions comes closest to how you feel about your household income nowadays?   ❑ Living comfortably on present income  ❑ Coping on present income  ❑ Difficult on present income  ❑ Very difficult on present income  ❑ Don´t know  ❑ Prefer not to answer   1. Has the physical condition or medical treatment of your partner/family member/friend with cancer caused you financial difficulties?   ❑ Not at all  ❑ A little  ❑ Quite a bit  ❑ Very much  ❑ Prefer not to answer  The following question concerns an entirely different topic. \| \| --- \|  1. Do you agree with the following statement: ´I believe in God or in someone or something greater than myself´.   ❑ Not at all  ❑ A little  ❑ Quite a bit  ❑ Very much    ❑ Prefer not to answer | | | | |  |
| 1. Your country of birth: ___________   If your mother or father was born in another country, please state it below:    Country of birth of mother: _______________  Country of birth of father: _______________ | | | | |  |
|  | | | |  | |
